# Hepatotoxicity due to chenodeoxycholic acid supplementation in an infant with cerebrotendinous xanthomatosis: implications for treatment

**DOI:** 10.1007/s00431-015-2584-7

**Published:** 2015-07-10

**Authors:** Hidde H. Huidekoper, Frédéric M. Vaz, Aad Verrips, Annet M. Bosch

**Affiliations:** Department of Pediatrics, Center for Lysosomal and Metabolic Diseases, Erasmus Medical Center-University Hospital, PO Box 2060, NL-3000 CB Rotterdam, The Netherlands; Department of Pediatrics, Academic Medical Center, University of Amsterdam, Amsterdam, The Netherlands; Department of Clinical Chemistry, Laboratory for Genetic Metabolic Diseases, Academic Medical Center, University of Amsterdam, Amsterdam, The Netherlands; Department of Neurology, Canisius Wilhelmina Hospital, Nijmegen, The Netherlands

**Keywords:** Cerebrotendinous xanthomatosis, Chenodeoxycholic acid, Treatment, Infant, Newborn screening, Hepatotoxicity

## Abstract

We present a two-week old girl who was diagnosed with cerebrotendinous xanthomatosis (CTX), an inborn error of bile acid synthesis, after a diagnostic workup for convulsions which were shown to be caused by a parechovirus encephalitis. The diagnosis of CTX was confirmed with *CYP27A1* mutation analysis. She was started on chenodeoxycholic acid (CDCA) supplementation, which inhibits cholestanol production through a feedback mechanism, at the advised dosage of 15 mg/kg/day. Within 6 weeks, she developed jaundice with hepatomegaly. CDCA supplementation was stopped after which liver size and function rapidly normalised. CDCA supplementation was then restarted and maintained at 5 mg/kg/day. Cholestanol, liver enzymes and total bilirubin were frequently monitored in the patient, who is now 2.8 years of age, and have remained within normal range. Her psychomotor development has been normal.

*Conclusion*: adequate metabolic control was achieved in an infant with CTX with CDCA supplementation at a dosage of 5 mg/kg/day and was well tolerated. CDCA supplementation at 15 mg/kg/day seems hepatotoxic in infants and should not be used. This is relevant in view of the possible inclusion of CTX in newborn screening programs in the near future.

**Table:** 

**What is Known:** • *Cerebrotendinous xanthomatosis (CTX), an inborn error of bile acid synthesis, is a progressive neurological disorder.* • *Symptoms of CTX can be halted, and likely prevented, with chenodeoxycholic acid (CDCA) supplementation, making CTX a good candidate for newborn screening.*
**What is New:** • *CDCA supplementation at the advised dosage of 15 mg/kg/day in children seems hepatoxic in infants with CTX*. • *Adequate metabolic control in an infant with CTX was achieved with CDCA supplementation at 5 mg/kg/day and well tolerated.*

## Introduction

**Fig. 1:**
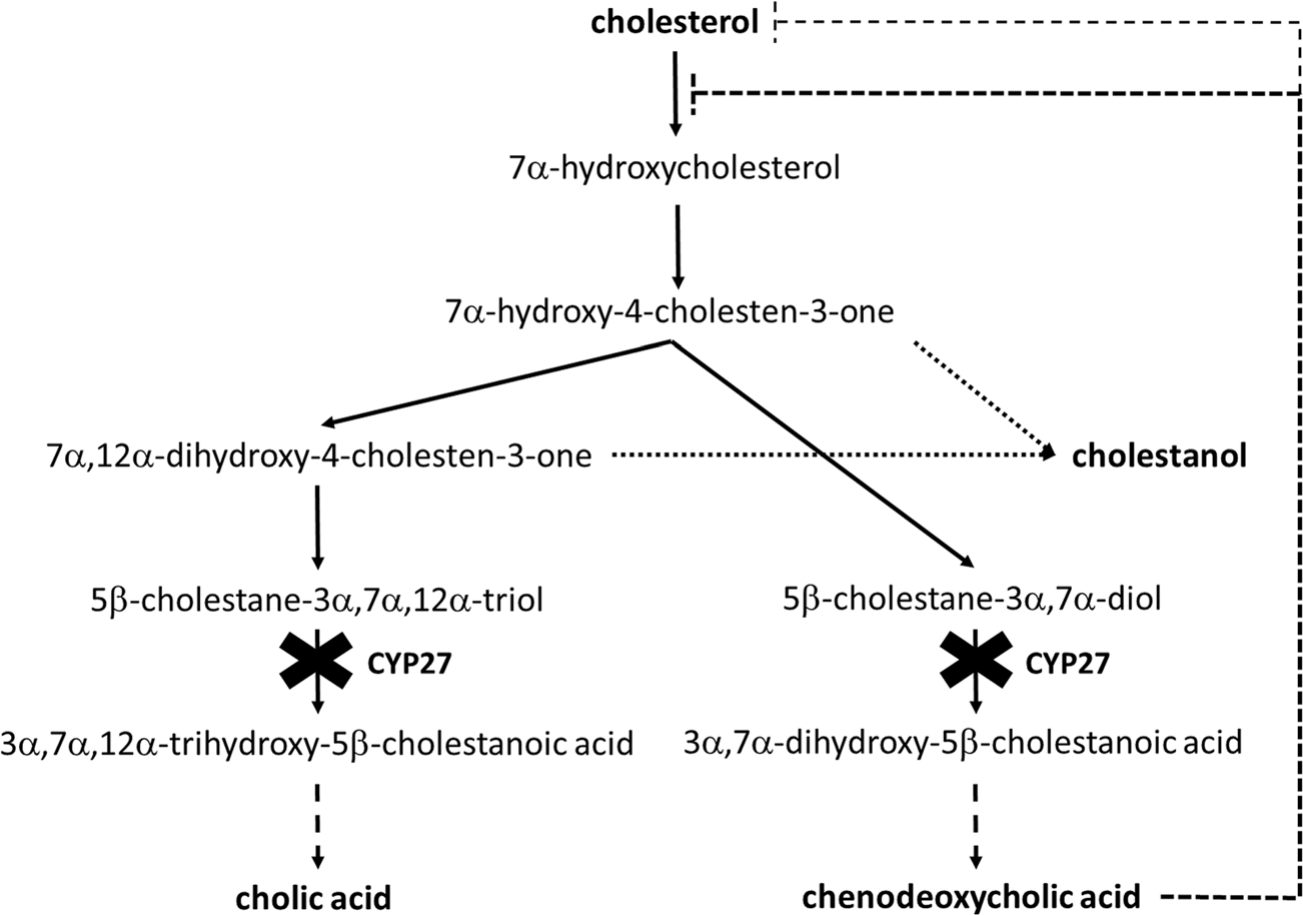
Bile acid synthesis in CTX. Due to the deficiency of 27-sterol hydroxylase (CYP27), the production of chenodeoxycholic acid (CDCA) and cholic acid (CA) is reduced and cholestanol and cholesterol accumulate. CDCA supplementation inhibits bile acid synthesis via negative feedback on both the cholesterol 7α-hydroxylase pathway and on cholesterol biosynthesis (*dashed lines*), thereby preventing cholestanol and cholesterol accumulation

Cerebrotendinous xanthomatosis (CTX) [OMIM #213700] is a rare autosomal recessive disorder of bile acid synthesis caused by the deficiency of the enzyme 27-sterol hydroxylase (CYP27) [EC 1.14.13.15]. This results in a reduced production of chenodeoxycholic acid (CDCA) and cholic acid (CA) and accumulation of cholestanol and cholesterol in different tissues (Fig. 1). Clinical features of CTX include neonatal cholestasis, bilateral cataract and chronic diarrhoea during childhood and tendon xanthomas and various neuropsychiatric symptoms, including pyramidal and cerebellar signs, peripheral neuropathy and dementia, from the second decade onward [10].

The development of these symptoms can be halted or prevented by supplementation of CDCA, which reduces bile acid synthesis via direct inhibition of the enzyme cholesterol-7α-hydroxylase (CYP7A1) [EC 1.14.13.17] and via negative feedback on cholesterol biosynthesis (Fig. 1) [5, 7]. The prognosis of CTX is good when therapy is started early but is less favourable when initiated at a later age [1, 9, 11]. Because early detection and start of treatment likely prevents symptoms, CTX is a good candidate for newborn screening. Although a validated screening method is not yet available, DeBarber et al. recently published a potential screening method for CTX based on the quantification of ketosterols [4].

The advised dosage of CDCA supplementation in children with CTX is 15 mg/kg/day [9]. Here, we describe an infant with CTX who developed toxic hepatitis with CDCA supplementation at this dosage and demonstrate that adequate metabolic control in infants and young children with CTX can also be achieved with the lower dosage of 5 mg/kg/day of CDCA. This is relevant in view of the possible inclusion of CTX in newborn screening programs in the near future.

## Case report

A girl, the second child of Dutch, non-consanguineous parents, was born at term after an uneventful pregnancy and delivery with a normal birth weight and normal Apgar scores. At day 8, she presented with feeding difficulties, lethargy and temperature instability, followed by convulsions for which continuous midazolam was started. No jaundice was present at this time. Initial workup, including full blood count, glucose, electrolytes, liver enzymes, coagulation, total bilirubin, C-reactive protein and blood gas analysis, only revealed prolonged coagulation times (PT and aPTT) which could be rapidly corrected with vitamin K. Further diagnostic workup, including blood, urine, faeces and cerebrospinal fluid cultures and viral PCR, a cerebral MRI and metabolic screening of plasma and urine, demonstrated a positive parechovirus PCR in cerebrospinal fluid, blood and faeces, consistent with the diagnosis of parechovirus encephalitis. Bilateral infarction of periventricular white matter without bleeding was shown on the cerebral MRI and was attributed to this encephalitis. After initial treatment on the pediatric intensive care unit, she was transferred to a pediatric ward, where she recovered without any signs of neurological sequelae. After 9 days, the girl was discharged in good clinical condition.

Coincidently, the metabolic screening demonstrated an elevated plasma cholestanol (21.5 μmol/L; reference range (age 0–100 days) 2.8–19.0 μmol/L), a low CDCA (0.1 mmol/L; reference range 0.7–10.0 μmol/L) and CA (0.4 mmol/L; reference range 0.1–4.7 μmol/L) and urinary excretion of the characteristic glucuronic acid conjugates of bile alcohols (cholestanetetrol, pentol and hexol), consistent with the diagnosis of CTX. No other metabolic abnormalities were detected. Mutation analysis of *CYP27A1* revealed two known missense mutations, c.1016C>T (p.Thr339Met) and c.1183C>T (p.Arg395Cys) [10], confirming the diagnosis of CTX. The patient was started on CDCA supplementation at the advised dosage of 15 mg/kg/day in three doses in children [9] resulting in the initial normalisation of plasma cholestanol (15.0 μmol/L), but 6 weeks after initiation of therapy, the patient presented with jaundice, pruritis and mild hepatomegaly (liver palpable 3 cm below right costal margin) without signs of infection. Total bilirubin (73 μmol/L), liver transaminases (ALT 336 U/L; AST 561 U/L) and alkaline phosphatase (647 U/L) were markedly elevated; gamma-glutamyltransferase (68 U/L) and coagulation times (PT 11.2 s; aPTT 20 s) were normal. Viral hepatitis due to an Epstein-Barr virus or cytomegalovirus infection was excluded. CDCA supplementation was stopped to rule out toxic hepatitis. Plasma CDCA concentration at this time was 467 μmol/L. After cessation of CDCA supplementation, liver size normalised within 1 month and liver enzymes returned to normal values within 3 months. CDCA supplementation was restarted at a dosage of 5 mg/kg/day, resulting in the normalisation of plasma cholestanol (reference range (age > 100 days) 3.5–10.0 μmol/L) and a plasma CDCA concentration between 6 and 12 μmol/L. The urinary excretion of CTX glucuronic acid conjugates of bile alcohols decreased to almost undetectable. The patient has been maintained on a CDCA dosage of 5 mg/kg/day since, and the plasma cholestanol concentration has remained within the normal range during 2.5 years of treatment. The patient’s growth and psychomotor development have been normal, and no clinical or neurological signs of CTX have been detected until now (she is currently 2.8 years).

**Fig. 2:**
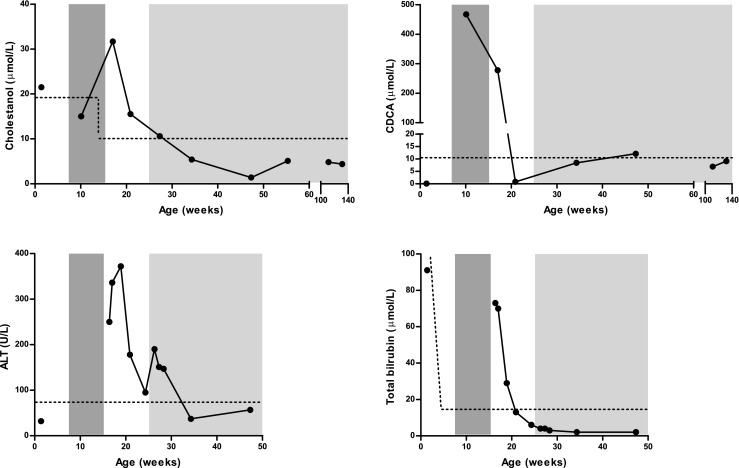
Plasma cholestanol (μmol/L), CDCA (μmol/L), ALT (U/L) and total bilirubin (μmol/L) at diagnosis of CTX (age 1.4 weeks) during treatment with CDCA supplementation at 15 mg/kg/day (dark shaded area), after cessation of CDCA supplementation and during CDCA supplementation at 5 mg/kg/day (light shaded area). The upper limit of normal for each metabolite is also shown (*dotted line*)

Plasma cholestanol, CDCA, ALT and total bilirubin concentrations before CDCA supplementation, during CDCA supplementation at 15 mg/kg/day and 5 mg/kg/day and in the period without treatment are shown in Fig. 2.

## Discussion

Cerebrotendinous xanthomatosis (CTX) is a well-established metabolic disorder of bile acid synthesis with serious progressive neurological sequelae without treatment. Supplementation of CDCA has been proven effective in mitigating the symptoms of CTX and will probably prevent the neurological phenotype when treatment is started at an early age [1]. Therefore, CTX is a good candidate for implementation in newborn screening programs. Currently, screening methods are being developed to make this feasible, and it is expected that CTX will be included in newborn screening programs in the near future.

In this report, we demonstrate the development of toxic hepatitis in an infant diagnosed with CTX shortly after birth after starting CDCA supplementation at the advised dosage of 15 mg/kg/day [9]. This is supported by the fact that liver size and function rapidly normalised after cessation of CDCA supplementation and that common causes for viral hepatitis in children were ruled out. Hepatitis A to E were not excluded, but it is highly unlikely that either one of these caused the hepatitis as neither the patient nor her mother did receive any blood products, hepatitis A and E are not endemic in The Netherlands, and no trips to foreign countries were undertaken. Furthermore, the potential hepatotoxic effect of CDCA, as well as other bile acids, is well established and has been shown to be dose dependent [8].

Although neonatal cholestasis is part of the clinical spectrum of CTX, it is unlikely that CTX by itself caused the hepatitis as our patient only developed jaundice after initiation of CDCA supplementation. However, the hepatotoxic effect of CDCA in our patient may have been aggravated by the accumulation of bile acid synthesis intermediates in hepatocytes. This would also explain the observed rise in plasma cholestanol with the development of hepatitis. In support of this is the observation that liver function deteriorated in an infant who presented with giant cell hepatitis and who was started on CDCA supplementation at a dosage of 10 mg/kg/day under suspicion of a bile acid synthesis defect [2]. He was later diagnosed with CTX [3].

It has been proposed that CTX in infants may be best treated with CA supplementation because of the potential hepatotoxic effect of CDCA [6]. However, we think that supplementation of CDCA is the right choice of therapy in CTX, also in infants, as it is a more potent stimulator of the nuclear receptor FXR, thereby effectively inhibiting cholestanol formation through the cholesterol 7α-hydroxylase pathway in CTX [5]. Here, we show that CDCA supplementation at a dosage of 5 mg/kg/day is safe and effective in an infant with CTX.

In this report, we demonstrate that adequate metabolic control in an infant with CTX was achieved with CDCA supplementation at a dosage of 5 mg/kg/day and was well tolerated. The currently advised dosage of 15 mg/kg/day of CDCA seems hepatotoxic in infants and should not be used in young children. This is relevant in view of the possible inclusion of CTX in newborn screening programs in the near future.

## Abbreviations

CTX: Cerebrotendinous xanthomatosis
CDCA: Chenodeoxycholic acid
CA: Cholic acid
